# Codes of ethics for psychiatrists: past, present and prospect

**DOI:** 10.1017/S0033291722000125

**Published:** 2022-05

**Authors:** Sidney Bloch, Felicity Kenn, Izaak Lim

**Affiliations:** 1 University of Melbourne, Melbourne, Australia; 2 Royal Australian and New Zealand College of Psychiatrists, Melbourne, Australia;; 3 Monash University, Melbourne, Australia

**Keywords:** Ethics, code of ethics, code of conduct, ethical standards, professional behaviour

## Abstract

**Background:**

Codes of ethics in medicine have an ancient tradition, extending back to the Oath of Hippocrates. Yet it was only in the early 1970s that the speciality of psychiatry developed a specific code to address the unique ethical dilemmas and complexities arising in psychiatric practice. As the 50^th^ anniversary of the publication of psychiatry's first code of ethics approaches, it is timely to reflect on the progress, role, and impact of such codes.

Our aim is to provide a historically informed review of codes of ethics in psychiatry – their origins and evolution, the current picture, and the possibilities for future development.

**Methods:**

We conducted a selective review of relevant literature (including all codes of ethics accessible on the websites of World Psychiatric Association members states), analysis of the form and content of codes and related documents in psychiatry, and interviews of psychiatrists who have played central roles in their evolution.

**Results:**

Of the 143 WPA member states, only 15 codes of ethics for psychiatrists were identified, and few of these were associated with professional disciplinary processes. We found that these codes are rarely revised and sometimes supplemented with other statements and guidelines.

**Conclusions:**

While there are difficulties in measuring the direct effectiveness of codes of ethics on the practice of psychiatrists, we conclude that these codes help to (1) promote professional solidarity and autonomy, (2) enhance moral sensitivity, and (3) aid in psychiatric education and training.

Psychiatry as a branch of medicine has been subject to codes of ethics indirectly for centuries but only in 1970 was it deemed desirable to produce a code for psychiatrists exclusively on the premise that many ethical problems they face ‘differ in colouring and degree from ethical problems in other branches of medical practice’ (Radden, 2002). Half a century later, it seems timely to offer a historical perspective of this core dimension of medical ethics. We have undertaken this task by reviewing relevant literature, scrutinising codes and related documents in psychiatry, and interviewing colleagues who have played central roles in their evolution.

We shall focus on the development and status of these codes, particularly the forms they have taken, the purposes they have served, and the types of influence they have had on the psychiatric profession. We will end with our views of their prospect in the light of our findings.

## In the beginning

Facing growing public concerns about the improper conduct of psychiatrists in the 1960s, the American Psychiatric Association (APA), took the unprecedented step in 1970 of devising a code of ethics for its members. Robert Moore (1978) observed that his colleagues had faced ‘a social egalitarian revolution demanding that the patient be given greater responsibility in determining his or her care and involving less willingness to accept the opinions of professional experts… [which] resulted in skyrocketing malpractice problems and growing complaints that psychiatry will not or cannot police itself.’

The committee was constrained by the American Medical Association's position that psychiatrists, as physicians, should be bound by its own *Principles of Medical Ethics* but was free to elaborate on them. Thus, comments referred to as ‘annotations’ were added to capture the essence of typical ethical challenges encountered in psychiatric practice. The result was the publication in 1973 of the *Principles of Medical Ethics with Annotations Especially Applicable to Psychiatry* (American Psychiatric Association, 1973). Its many revisions have all adhered to the original format, embedding psychiatric ethics within the orbit of general medical ethics (P. Appelbaum, personal communication, 25 October 2020).

At an international level, the most momentous development in parallel with the APA initiative, was the World Psychiatric Association's (WPA) focus on the ethical domain for the first time. Although the WPA was initially sceptical about mounting evidence of the former Soviet Union's alleged misuse of psychiatry to suppress political and religious dissent, several member societies entreated it to investigate the allegations and to create a code to uphold high standards of ethical conduct (Bloch & Reddaway, 1977, 1984).

Clarence Blomquist (1977), a prominent Swedish ethicist, served as a consultant in drafting a suitable document. He regarded prevailing medical codes as unsuitable given their paternalistic quality and inattention paid to the public interest. Blomquist's proposals ultimately prepared the ground for the 1977 *Declaration of Hawaii* (Ottosson, 2000). As new ethical dilemmas emerged, the need to update the document became obvious. In 1996, a revised edition, newly entitled the *Declaration of Madrid*, introduced guidelines to deal with issues such as euthanasia, torture, and the death penalty (Helmchen & Okasha, 2000).

The European Psychiatric Association (EPA) was the first regional psychiatric organisation to produce an ethically oriented document in its *Declaration on Quality of Psychiatry and Mental Health Care in Europe* in 2013 (European Psychiatric Association, 2013); the EPA regarded its 10 statements as requisites to safeguard the quality of care and called for their endorsement.

In 1991, the United Nations (UN) manifested its concern about the lack of rights accorded to the mentally ill. The outcome was *Principles for the Protection of Persons with Mental Illness and the Improvement of Mental Health Care* (UN General Assembly, 1991). States were urged to enact measures (e.g. legislative, administrative, and educational) to advance objectives such as treating patients with respect, providing them with the best attainable care and using the least restrictive setting. The necessity to treat some patients involuntarily was acknowledged but only when two provisos were satisfied: the procedure had to meet explicit criteria and a psychiatrist's recommendation for its deployment had to be independently reviewed.

A sequel to the *Principles* was the *UN Convention on the Rights of Persons with Disabilities* (UN General Assembly, 2007) with its emphasis on States to protect ‘all human rights and fundamental freedoms’ of people with any psychosocial disability including the mentally ill ‘and to promote respect for their inherent dignity’.

Returning to national codes, it is striking how so few associations of psychiatry have imitated the APA over five decades, although a proportion has selected other means to uphold ethical practice (see below). Organisations that have produced a code have been clearly influenced by distinctive local factors as reflected in the following examples.

The Royal Australian and New Zealand College of Psychiatrists (RANZCP) published its code in 1992 in response to a series of sexual boundary violations and to unethical conduct in two psychiatric services, all of which had markedly tarnished the profession (Anderson, 1991; Wilson, 2003). The code was one of several components produced to improve standards of practice and to prevent misconduct (Pargiter & Bloch, 1994; Rubinstein, 1996).

The Russian Society of Psychiatrists also produced a code in the early 90's albeit in the context of dramatic circumstances. The dissolution of the former Soviet Union in 1991 had led to a radical transformation of psychiatry which had been hierarchical rigidly controlled by the State for decades. The code symbolised the advent of a new era; psychiatrists felt empowered to function free of political interference (Polubinskaya & Bonnie, 1996).

The Japanese Society of Psychiatry and Neurology's (JPN) motivation to devise a code also had unique features. In the aftermath of Japan's defeat in the Second World War, the Government began to subsidise private hospitals, many owned by psychiatrists, in a desperate effort to provide care for thousands of homeless mentally ill patients. The system was ripe for abuse since the longer patients were hospitalised, the greater were the profits (Harding, 1991). Many years elapsed before this arrangement was supplanted by a community-based system of care. The publication of a Japanese translation of a textbook on psychiatric ethics in 2011 saw the formation of an ethics committee, and the compilation of 14 moral principles. A comprehensive code was soon deemed necessary, but the task has remained under consideration (C. Fujii, personal communication, 30 August 2020).

In several associations, a delay, for a range of reasons, has occurred prior to taking the plunge. For instance, the case for a code for the British Royal College of Psychiatrists was first mounted in 2003 when two members argued that their colleagues needed to ‘define (their) ethical identity… based on a system of values that are clinically meaningful and respectful of diversity.’ (Sarkar & Adshead, 2003) The code materialised but only following a protracted exchange about what should constitute its *raison d'etre*. An assiduous examination of codes of other psychiatric associations proved most helpful (see below) (G. Adshead and M. Deshpande, personal communication, 3 June 2020).

The College of Psychiatrists of Ireland by contrast formulated *Professional Ethics for Psychiatrists* in a prompt response to a request by the Medical Council of Ireland to its constituent medical organisations to devise their own codes, in recognition of the need to promote ethical practice (College of Psychiatrists of Ireland, 2019).

## Alternative approaches to promote ethical practice

Many associations have resorted to means other than devising a code in order to raise ethical standards.

Reliance on ethical instruments of related organisations, particularly the WPA's *Declaration of Madrid*, has been the most common strategy. This makes sense given that the *Declaration* is ‘owned’ by all its member societies; moreover, it probably has suited those in middle- and low-income countries where resources needed to produce a code have been limited.

But a snag prevails in using a code of this type; a global organisation faces the hurdle of taking account of local factors such as mental health law and social and cultural norms. Furthermore, the procedure of regularly revising a code, especially when aiming for consensus, is bound to be unwieldy. The WPA's *Declaration* has been viewed by some psychiatrists well informed about ethics as a nebulous ‘aggregate of statements’, especially with regard to assessing a member society's alleged ethical misconduct (P. Appelbaum, personal communication, 25 October 2020).

On the other hand, a group of senior WPA psychiatrists were reluctant to relinquish a document they believed had proved its worth.

Nevertheless, a new code comprising principles and annotations, similar in format to several national codes, was developed over several years, and finally adopted in October 2020 with the *Declaration* remaining as an adjunct (World Psychiatric Association, 2020). To circumvent the problem of legal differences prevailing among the jurisdictions of member societies, a qualification ‘in accordance with local law’ was cited where necessary. The authors of the WPA *Code* acknowledged that many societies had produced their own codes but nonetheless requested them to endorse the principles of the new document and to use them as a guide when revising their own code (Appelbaum & Tyano, 2021).

Many psychiatric associations have utilised an existing code produced by the State's medical profession as a whole. The Israel Psychiatric Association (IPA), for example, has always subscribed to the Israel Medical Association's (IMA) code even though not all psychiatrists have found the arrangement satisfactory in that it has not done justice to ethical challenges unique to their practice. In 2016, a group within the IPA proposed that their association devise its own code; however, another group were not in favour since this would impose additional obligations on psychiatrists already subject to the IMA code. (R. Strous, personal communication, 9 July 2020). Ironically, in a survey of Israeli psychiatrists at the time, most admitted that their knowledge of ethics was scanty (Bergman-Levy et al., 2016).

In Nepal, all doctors, including psychiatrists, have had to swear an oath to comply with the Nepal Medical Council's *Code of Ethics*. Although psychiatrists have been encouraged to use the *Declaration of Madrid* as well, they have stated, with admirable candour, that imperfect mental health legislation and the lack of a specific code for themselves, have contributed to ethical shortcomings in their practice (Upadhyaya & Joshi, 2011).

## The current picture

In order to ascertain the place of codes in contemporary psychiatry, we have scrutinised the websites, where available, of the 143 member societies of the WPA (Table 1). The utility of this approach has been limited by a number of societies not having a website and others not mentioning the existence of, or providing a link to, a code in their website. Where possible we have tried to contact such societies directly.

**Table 1.** Prevalence of ethical resources in 143 member societies of the World Psychiatric Association

Type of ethical resource	Number
Has explicit code of ethics^a^	15
Uses the code of a universal or regional psychiatric organisation e.g., the WPA Declaration of Madrid, European Psychiatric Association	8
Uses the code of a national general medical association e.g., Israel Medical Association	7
States that ethics is central to psychiatric practice but does not elaborate	11
Has an ethics committee but no code cited	9
Uses guidelines on specific ethical issues in psychiatry but has no code	6
Has a website but does not refer to any ethical resources	46
Has no website or any other online presence (although it is possible that ethical aspects of practice do exist)	41

a American Psychiatric Association (including Puerto Rican Society of Psychiatry), Argentinean Association of Psychiatrists, Armenian Psychiatric Association, Canadian Psychiatric Association, College of Psychiatry of Ireland, Colombian Association of Psychiatry, Estonian Psychiatric Association, French Association of Psychiatrists in Private Practice, Hungarian Psychiatric Association, Japanese Society of Psychiatry and Neurology, Netherlands Psychiatric Association, Peruvian Psychiatric Association, Royal Australian and New Zealand College of Psychiatrists, Royal College of Psychiatrists (UK), Russian Society of Psychiatrists.

The 15 codes we have identified vary considerably in terms of the degree to which they are based on moral concepts. Most commonly, they contain overarching moral principles and a series of annotations accompanying each one which elaborate on their meaning, point out how they are deployed, and illustrate that even a lofty principle (together with its annotations) cannot always resolve a moral conundrum such as when two relevant principles clash with each other.

Many ethical codes have a hybrid character since they comprise both morally based principles and features more typical of a ‘code of practice’ (also referred to as a ‘code of conduct’) in which a set of explicit rules predominate. For example, the Colombian Psychiatric Association's code comprises a set of principles, among them integrity, ‘character of correctness and goodness’ and trustworthiness as well as guidelines on pragmatic aspects of clinical practice such as advertising and setting fees (Asociación Colombiana de Psiquiatría, 2006). Similarly, the Argentinian Association of Psychiatrists' code has three sections: ‘ethical principles’ (e.g. respect, confidentiality and consent), ‘vices a psychiatrist should not incur’ (e.g. abuse, negligence), and rules-based ethics such as not falsifying professional records (Asociación Argentina de Psiquiatras).

The APA code amalgamates ethically informed principles with pragmatic rules as exemplified in Section 1. The principle calls on psychiatrists to provide competent medical care with respect for human dignity and not to exploit the patient. A later section covers rules related to fees for missed appointments and splitting of fees in specified circumstances.

While clearly influenced by various theoretical traditions (e.g. utilitarianism, deontology, virtue ethics, principlism), none of the codes we reviewed has a specific, unifying theoretical commitment, but instead are pragmatic composites that have emerged from the profession itself.

## Revising codes

A pertinent matter when devising a code is how and when to revise it. A code is ideally a dynamic document whose ‘custodians’ strive to optimise its utility and relevance in the light of changing clinical practice, scientific advances, and societal developments, especially legal and legislative (Brotherton, Kao, & Crigger, 2016). The rationale for changes is made explicit in some codes but barely mentioned in others. Examples of the former are the Irish College which states that its code ‘may be subject to change… to reflect changing societal attitudes and norms, and changes in relevant legislation’ and the RANZCP which informs that its code ‘is regularly revised and updated [in fact, every 5 years] to ensure that it remains relevant and contemporary.’ The Netherlands Psychiatric Association has updated its code in conjunction with amendments to the Royal Dutch Medical Association's code but states that this could occur at other times if the need arose (Nederlandse Vereniging voor Psychiaters, 2010).

As far as we can ascertain, only a minority of codes have been revised, with only four out of a possible fifteen being identified (American Psychiatric Association; Canadian Psychiatric Association; Netherlands Psychiatric Association; Royal Australian and New Zealand College of Psychiatrists). Impediments may derive from an assumption that moral principles are enduring or from perceived difficulties in determining how to expedite the task or that more recently established codes do not yet warrant a change. Associations which have not revised their code could benefit from examining those undertaken by sister organisations and seeking their advice on how to go about it. In addition, the latter could publish the changes made and their rationale (Bloch, Kenn, & Smith, 2018; Lim, Kenn, & Bloch, 2022; Steinberg, Bloch, Riley, & Zelas, 2000).

## Do psychiatric sub-specialties need their own codes?

The argument could be advanced that certain types of psychiatric practice present ethical challenges *sui generis*. Psychogeriatrics, child and adolescent psychiatry, community psychiatry and forensic psychiatry spring to mind. Our review however has yielded a minute number of codes targeted to these sub-specialities, arguably on the grounds that a general psychiatric code is regarded as sufficiently all-embracing. Our findings suggest the opposite. A couple of examples make the point: the RANZCP code contains only one annotation pertaining to child and adolescent psychiatry and none to forensic psychiatry; the APA code also only has a single reference to children and adolescents, and only two to the forensic domain.

We have found two noteworthy codes devised for sub-specialities, one for child and adolescent psychiatrists (CAPs), the other for forensic psychiatrists. Both were conceived by American academies in these fields. They warrant attention since they could serve as models for psychiatrists in other sub-specialities should they deem it advantageous to have a code germane to their practice.

During the 1960s and early 1970s, legal and legislative landmarks concerned with the maltreatment of children came to the fore in the United States. The American Academy of Child and Adolescent Psychiatry (AACAP) saw the need for a code to deal with these issues. Setting up an ethics committee and publishing a code ensued in 1980 (Sondheimer & Klykylo, 2008). The principal goal of the latter was to highlight CAPs' obligation to promote the care of patients (and their families/guardians) by specifying required professional standards and the range of ethical problems of which they should be cognisant; the latter included, *inter alia*, the inherent vulnerability of children and adolescents, obligations to protect them from harm and to advocate on their behalf, their restricted capacity to give informed consent, and participation in their care of multiple parties including parents or guardians, teachers, child protection agencies and paediatricians.

The AACAP has also been exemplary in keeping abreast of new ethical developments. Thus, the code has been updated periodically (A.Sondheimer, personal communication, 25 July 2020). Unusually, Academy members can propose an amendment at any time as well as seek the ethics committee's advice about a concern they have (commendably, other health care professionals and the laity can also request advice).

The ethically oriented activities undertaken by the American Academy of Psychiatry and the Law (AAPL) is equally meritorious. The organisation, established in 1969, immediately set up an ethics committee to tackle a range of concerns (Weinstein, 1984). When a plan to add ethical guidelines to the APA code relevant to forensic psychiatry did not make any headway, the Academy promptly produced its own code; revisions have followed over the years. Interestingly, the term ‘guideline’ was preferred to ‘principle’ for fear that a member might face a lawsuit consequent on an alleged failure to adhere to explicit standards of practice.

Appelbaum (1997) posits that there is a clear distinction between clinical and forensic roles, stressing that psychiatrists who conduct evaluations for legal purposes do not enter into a physician-patient relationship, and therefore the ethical principles that apply in the latter situation differ from those in the former.

Conceptual hurdles, it would seem, can vitiate the construction of a sub-speciality code. A group of British forensic psychiatrists, using two representative cases to illustrate ethical dilemmas they had encountered, assumed that marshalling their professional experience would shed light on the type of code they required (Sen, Gordon, Adshead, & Irons, 2007). Although a consensus was reached that the principle of justice should take precedence over other principles, a code did not materialise.

## Should a code have ‘teeth’?

We have found that associations differ on whether their code should encompass a sanctioning role in the event of an alleged breach of one or more of its principles.

Very few associations (APA, Argentinean Association of Psychiatrists and Colombian Association of Psychiatry) appear to have deployed their code to investigate a member's alleged unethical conduct. The APA is a notable exception: when a complaint is lodged, the local branch ethics committee clarifies whether it has violated a principle of the code. If the complaint is upheld, disciplinary action ranging from admonition to expulsion ensues. A national ethics committee undertakes a more extensive investigation in the case of a serious violation.

Paul Appelbaum (personal communication, 25 October 2020), a former President of the APA, has contended that an association-based inquiry is both cumbersome and costly; moreover, an all-psychiatrist panel is apt to be subject to bias as well lack expertise in ensuring due process, both factors vitiating a fair hearing. The RANZCP initiated a procedure like that of the APA but concluded before long that it was indeed labour intensive and due process was questionable; the task was then transferred to a statutory body whose final judgement was carefully appraised by the College to determine what action, if any, it should take.

Some associations have adopted a rather ambiguous position. For instance, the Royal College of Psychiatrists' code explicitly stipulates that although it ‘does not have the force of law… principles must be adhered to without variation or exception’ (Royal College of Psychiatrists, 2014) while the RANZCP's code stipulates that ‘[it] may be applied by other bodies as a benchmark of satisfactory ethical behaviour’ and ‘does not release psychiatrists from the obligations and responsibilities laid upon them by other recognised ethical statements.’ (Royal Australian & New Zealand College of Psychiatrists, 2018a)

## The role of ‘position statements’

Many psychiatric associations have understood that new ethical issues surface periodically which a code has not, or only tangentially, addressed (Pargiter & Bloch, 1997). If a revision is not due for some years, issuing a timely ‘position statement’ (PS) has been commonly expedited. Indeed the RANZCP code's preamble highlights that the Code ‘should be …used in the context of other RANZCP resources such as PSs and clinical practice guidelines.’

If the resultant PS remains relevant, it may be woven into the code's next edition. Although most PSs deal with matters in the sphere of the State rather than ethical issues individual psychiatrists can address, there is a potential overlap. Of the many PS's we have come across in our review, the following examples will suffice to convey their typical features with respect to the overlap.

The EPA issued a position paper in 2015 to draw European policy makers' attention to the urgent need of refugees for appropriate mental health care. Among its eight recommendations were improving access to treatment, adopting a culturally sensitive approach, and educating health care professionals for this type of work (European Psychiatric Association, 2015).

In a similar vein, the RANZCP produced a statement in 2018 highlighting the urgent mental health needs of child asylum seekers (Royal Australian & New Zealand College of Psychiatrists, 2018b). Among its key points were their right to equal access to healthcare, particularly specialist treatment, given their experience of severe trauma in the past, prolonged detention, and an uncertain future. College members were also urged to advocate on the children's behalf by highlighting the State's obligations. A related document points out the capacity of psychiatrists to provide high-quality mental healthcare and to practice ethically and effectively in detention centres and alternative places of detention is limited and provides guidance on negotiating conflicts of interest that arise when the responsibility of psychiatrists to their patients may be incompatible with those to governmental authorities (Royal Australian & New Zealand College of Psychiatrists, 2016).

The Royal College of Psychiatrists has supplemented its code with PSs on such contemporary matters as the COVID-19 pandemic. Members are reminded of their crucial role in not only caring for patients but also supporting medical colleagues who are working at the front line (Royal College of Psychiatrists, 2020).

The APA issued a PS in 2018 (American Psychiatric Association, 2018) opposing racism and racial discrimination and stressing their adverse impact on the mental health and wellbeing of people of colour as well as of the nation as a whole. *Inter alia*, it supports actions that psychiatrists can undertake to promote multiculturalism, diversity, advocacy for equitable services and policies to reduce further harm.

## Are codes effective?

A claim made by several critics is the failure of codes to boost the standard of moral behaviour (Bowie, 1979; Ladd, 1985; Morin, 2005). A counterargument has prevailed, namely that codes are potentially effective according to the purposes its creators have set. We have explored this key topic by attempting to detect outcomes that associations have sought to achieve.

A code may theoretically influence professional conduct directly when psychiatrists are cognisant of its purpose/s and consult its principles and annotations whenever they wrestle with an ethical conundrum. Identifying indirect effects has proved more elusive but appears to include psychiatrists embracing the code as a moral compass for pressing contemporary socio-political issues such as the deleterious impact of ‘hate speech’ in the social media, the plight of detained asylum seekers, and a pandemic's adverse effects on mental health.

Empirical studies to measure a code's effectiveness in curtailing misconduct, whether directly or indirectly, are conspicuously absent. The reason is not hard to deduce – how does one distinguish the impact of a code from myriad other factors that influence ethical decision-making? Not too surprisingly, the meagre research reported has yielded mixed findings. For example, most participants in a study of nurses' attitudes to codes were unfamiliar with the content of their own codes and saw them as of little practical value (Tadd et al., 2006). Negative comments of a similar kind have been found in qualitative analyses of other nursing samples (Heymans, van der Arend, & Gastmans, 2007). Paradoxically, the former sample regarded codes as an asset for forging a professional identity and affirming shared ethical values to foster an image of the ‘ideal professional.’

A survey of physicians' views yielded similar results: limited awareness of their code, negligible impact on practice, marginal utility, and less relevant than State law and policy (Malloy et al., 2009). However, sub-group thought codes were of utility with the reservation that ethical decision-making was also influenced by personal and cultural values.

Systematic studies of the views of psychiatrists are sorely needed.

## Whither codes in psychiatry?

Our first step in considering the future of psychiatric codes is to cite the views of three academics whose lines of reasoning are impressively coherent.

Judith LIchtenberg (1996) uses a utilitarian argument when postulating that a code ‘increase[s] the likelihood that people will behave in certain ways, partly by bringing to consciousness the character of their actions, partly by attaching sanctions to non-compliance, and partly by increasing the value of… an individual act.’ She concludes that ‘ethics is only partly concerned with acting for the right reasons…; it is also concerned with getting people to behave in ways that have been determined… to be morally desirable or required.’ By a process of extrapolation, we posit that a motivated group of psychiatrists can jointly identify principles to enhance their moral integrity.

Ruth Macklin (2015) complements Lichtenberg's points in asserting that ethical decision-making is a reflective, rational, and deliberative process which relies on the quality of analysis and ethical justification in tandem with coherent and explicit principles. Although not referring to codes specifically, she alludes to them when suggesting that a catalogue of moral principles is invaluable to guide one's thinking when wrestling with an ethical quandary.

For Sarah Banks (2003), a code serves rhetorical, educational, and regulatory functions. Notwithstanding that its principles, however comprehensive and judiciously selected, cannot encompass all contingencies, they can still offer professionals a foundation on which to pursue their tasks at the highest possible standard. This is especially the case when they have shared the responsibility of creating the code *ab initio*. They can also draw on their conscience, exchanges with colleagues, and values espoused by their organisation.

A second step in responding to the question of whether codes derive from the findings of our review. We are struck by three features of a code which we suggest augurs well for its future:

- Members of an association are bound to benefit by sharing the responsibility to devise (and revise at regular intervals) a code with the objective of promoting a united sense of professional autonomy. Instead of being subject to the whims of a faceless bureaucracy (such as a licensing authority) which dictates how psychiatrists should conduct their affairs, the association could govern itself with the code reflecting its values and aspirations. The code could also symbolise the members' commitment to advance an ethos of mutual respect and justice and to disseminate that ethos to the people who depend on their expertise, as well as to the community at large.
- A code could play a key part in enhancing the moral sensitivity of an association's members in at least two ways: by alerting them to ethical quandaries inherent in their work and inspiring them to cultivate a keen ‘clinico-ethical consciousness’. By moral sensitivity, we refer to a state of receptiveness in which professionals turn to moral principles in ‘real time’ when facing a demanding ethical encounter. For example, a psychiatrist may recognise that a feature of the code resonates with their experience of such repeated challenges as treating patients compulsorily, attempting to prevent them from suicide and obtaining informed consent in the case of limited capacity. Importantly, the self-awareness and critical reflection associated with moral sensitivity allow clinicians to identify the sources and severity of ‘moral distress’, which is a vitally important step in addressing the detrimental impact of such an experience (Moffat, 2014; Rushton, 2006).
- A code operating as an educational resource enables practitioners to appreciate that ethical challenges are subject to systematic analysis through applying rational argument and justifying its premises (as Macklin argues above). Annotations are particularly effective by demonstrating that even hallowed principles may resist application such as when they collide with one another. A code also could offer trainee psychiatrists the opportunity to acquaint themselves with concepts in moral philosophy which underlie ethical decision-making (complemented by a bibliography) and, by so doing, advance requisite skills

A code merits a prominent place in training from the outset; novices after all may encounter an ethical challenge on their very first day! A copy of their association's code could be ceremoniously presented to them to symbolise how essential it will be throughout their career.

The code could also feature in the association's continuing professional development programme and academic conferences. Contributors to an ‘Ethical Matters’ column in the association's newsletter could reference the code when responding to members' queries. The APA has pursued a similar practice whereby its Ethics Committee issues what it refers to as ‘ethical opinions’ in response to members' queries about ethical quandaries and *pari passu* promote their understanding of the APA *Principles* and their applicability (American Psychiatric Association, 2017).

To further the sense of all members ‘owning’ their code and to keep ethical issues in the foreground, they could be encouraged to suggest amendments or additions.

Finally, the research could be conducted on such key topics as members' appraisal of a code's place in their practice, its effectiveness, and the rationale underlying any revision.

## Conclusion

We hope that our review will encourage psychiatric associations without a code to entertain the prospect of devising one. They need not feel daunted since they will not have to start from scratch. On the contrary, the structure and content of prevailing codes could serve as a model; moreover, their creators could offer advice. Smaller associations with insufficient resources could consult the WPA's *Declaration of Madrid* and the newly published code of ethics.

We are confident that psychiatrists would gain immeasurably were they to regard a code as pivotal to the welfare of patients, their families, and society overall. Fidelity to consensually derived ethical norms would also benefit psychiatrists themselves by bolstering their sense of solidarity and *esprit de corps* (Pellegrino, 1982), thus building a ‘moral community’ that confers protection against the damaging effects of moral distress (Traudt, Liaschenko, & Peden-McAlpine, 2016).

## Supplementary material

For supplementary material accompanying this paper visit https://doi.org/10.1017/S0033291722000125. click here to view supplementary material
