# Supplementary material for: Codes of ethics for psychiatrists: past, present and prospect
Source: Psychol Med. 2022 Feb 8;52(7):1201–7. doi: 10.1017/S0033291722000125 (PMC9157294; doi:10.1017/S0033291722000125)
Supplement: Supplementary file 1 [file S0033291722000125sup001.pdf]

Click here to access/download;Other Supplementary Material;Codes of ethics table supplement

Codes of Ethics: prevalence and form of ethical resources of WPA member societies

The tables below includes information on all member societies of the World Psychiatric Association (WPA), following website reviews, to determine which societies have a code of ethics or other ethical resources. It is noted that some societies would fit into more than one category (e.g. it may have guidelines on specific ethical issues and and ethics committee, but have no code of ethics). Societies have been placed in the category most relevant. These table were developed followed web-based search methodologies for each society, using supplementary literature where relevant. Supplementary Table 2 provided a summary of findings and supplementary Table 3 the full picture

Supplementary tables

Table 2: Summary table

**Type of code/ethical guidance**	**Society**	Total number
Full code of ethics stated on website	American Psychiatric Association<br>Argentinean Association of Psychiatrists<br>Armenian Psychiatric Association<br>Canadian Psychiatric Association<br>College of Psychiatry of Ireland<br>Colombian Association of Psychiatry<br>Estonian Psychiatric Association<br>French Association of Psychiatrists in Private Practice<br>Hungarian Psychiatric Association<br>Japanese Society of Psychiatry and Neurology<br>Netherlands Psychiatric Association<br>Peruvian Psychiatric Association<br>Royal Australian and New Zealand College of Psychiatrists<br>Royal College of Psychiatrists (UK)<br>Russian Society of Psychiatrists	15
Uses the code of an international or regional psychiatric organisation e.g., World Psychiatric Association, European Psychiatric Association	Puerto Rican Society of Psychiatry<br>Finnish Psychiatry Association<br>Psychiatrists' Association of Nepal<br>Ethiopian Psychiatric Association<br>Kenya Psychiatric Association<br>Lithuanian Psychiatric Association	6

Uses the code of a national general medical association e.g., Israel Medical Association	Barbados Association of Psychiatrists<br>Jamaica Psychiatric Association<br>Brazilian Association of Psychiatry<br>Norwegian Psychiatric Association<br>Israeli Psychiatric Association<br>Kuwait Psychiatric Association	6
States that ethics is central to psychiatric practice but does not elaborate	Guatemalan Psychiatric Association<br>Austrian Society for Psychiatry and Psychotherapy<br>German Association for Psychiatry, Psychotherapy and Psychosomatics<br>Saudi Psychiatric Association<br>Association of Psychiatrists in Nigeria<br>Uganda Psychiatric Association<br>Pakistan Psychiatric Society (education mission)<br>Bangladesh Association of Psychiatrists<br>Malaysian Psychiatric Association<br>Korean Neuropsychiatric Association<br>Taiwanese Society of Psychiatry	11
Has an ethics committee but no code cited	Flemish Psychiatric Association (Belgium)<br>Danish Psychiatric Association (also treatment guidelines)<br>Psychiatric Association of Turkey<br>Bulgarian Psychiatric Association<br>Slovak Psychiatric Association<br>Association of Neurologists, Psychiatrists and Narcologists of Ukraine<br>Philippine Psychiatric Association<br>The Hong-Kong College of Psychiatrists (China)	8
Uses guidelines or resources on specific ethical issues in psychiatry but has no code	Albanian Psychiatric Association<br>Singapore Psychiatric Association<br>Icelandic Psychiatric Association<br>Lebanese Psychiatric Society<br>South African Society of Psychiatrists<br>Spanish Society of Psychiatry<br>Romanian Association of Psychiatry and Psychotherapy	7
Has a website but does not refer to any ethical resources	Salvadorean Association of Psychiatry<br>Honduran Society of Psychiatry<br>Mexican Psychiatric Association	50

	Mexican Society of Neurology and Psychiatry<br>Panamanian Society of Psychiatry<br>Association of Argentinean Psychiatrists (APSA)<br>Foundation for Interdisciplinary Investigation of Communication (FINTECO) (Argentina)<br>Bolivian Society of Psychiatry<br>Psychiatric Association of Rio de Janeiro State (Brazil)<br>Society of Neurology, Psychiatry and Neurosurgery (Chile)<br>Paraguayan Society of Psychiatry<br>Society of Psychiatry of Uruguay<br>Royal Society of Mental Medicine of Belgium<br>French Association of Psychiatry<br>French Psychiatric Information Society<br>International Society of Psychopathology of Expression (France)<br>Medical Psychologic Society (France)<br>The Psychiatric Evolution (France)<br>Swiss Society of Psychiatry<br>Latvian Psychiatric Association<br>Swedish Psychiatric Association<br>Hellenic Psychiatric Association<br>Hellenic Society of Neurology and Psychiatry<br>Italian Psychiatric Association<br>Italian Association for Research in Schizophrenia<br>Portuguese Association of Psychiatry<br>Portuguese Society of Psychiatry and Mental Health<br>Spanish Association of Neuropsychiatry<br>Turkish Neuropsychiatric Society<br>Psychiatric Association of Bosnia-Herzegovina<br>Croatian Psychiatric Association<br>Czech Psychiatric Association<br>Polish Psychiatric Association<br>Association of Free Psychiatrists of Romania<br>Serbian Psychiatric Association<br>Association of Psychiatric Institutions of Serbia – UPUS<br>Psychiatric Association of Slovenia<br>Belarusian Psychiatric Association<br>Ukrainian Psychiatric Association	

	Association of Psychiatrists of Uzbekistan<br>Algerian Psychiatric Association<br>Egyptian Psychiatric Association<br>Syrian Arab Association of Psychiatrists<br>Iranian Psychiatric Association<br>Indian Psychiatric Society<br>Indian Association for Social Psychiatry<br>Indonesian Psychiatric Association<br>Sri Lanka College of Psychiatrists<br>The Psychiatric Association of Thailand<br>Chinese Society of Psychiatry	
Has no website or any other online presence (although it is possible that ethical aspects of practice do exist)	Costa Rican Psychiatric Association<br>Cuban Society of Psychiatry<br>Dominican Society of Psychiatry<br>Nicaraguan Psychiatric Association<br>Ecuadorian Association of Psychiatry<br>Venezuelan Society of Psychiatry<br>Luxembourguese Society of Psychiatry, Neurology and Psychotherapy<br>Cyprus Psychiatric Association<br>Macedonian Psychiatric Association (Republic of North Macedonia) (formerly: Psychiatric Association of Macedonia (FYROM))<br>Montenegrin Psychiatric Association<br>Society of Georgian Psychiatrists<br>Azerbaijan Psychiatric Association<br>Kazakh Association of Psychiatrists & Narcologists<br>The Association of specialists working in the field of mental health (Kazakhstan) (Ad-Hoc)<br>Kyrgyz Psychiatric Association<br>League for Mental Health from Republic of Moldova<br>Libyan Association of Psychiatry, Neurology and Neurosurgery 60<br>Sudanese Association of Psychiatrists<br>Moroccan Society of Psychiatry<br>Tunisian Society of Psychiatry<br>Arab Gulf Psychiatric Association<br>Emirates Society of Mental Health<br>Iraqi Society of Psychiatrists	39

	Jordan Association of Psychiatrists<br>Palestinian Psychiatric Association<br>Yemen Psychiatrists & Neurologists Association<br>Ghana Psychiatric Association<br>Society of Psychopathology and Mental Hygiene of Dakar (Senegal)<br>Botswana Psychiatric Association<br>Mauritius Psychiatric Association<br>Mozambican Association of Psychiatry and Mental Health<br>Zimbabwe College of Psychiatrists<br>Zambia Psychiatric Association<br>Afghanistan National Psychiatrist Association (ANPA)<br>Myanmar Medico-Psychological Society<br>Mental Health Association of Cambodia (MHAC)<br>Mongolian Mental Health Association<br>Papua New Guinea Psychiatric Association	

Supplementary table 3 – complete results

	**Organisation**	**Organisation website**	**Code of ethics information**
		Region 1: The Americas	
1	American Psychiatric Association	American Psychiatric Association | psychiatry.org	Has a code of ethics: American Psychiatric Association The Principles of Medical Ethics With Annotations Especially Applicable to Psychiatry (2013). Previous editions 1973, 1978, 1981, 1984, 1985, 1989, 1992, 1993, 1995, 1995 Revised, 1998, 2001, 2001 2006, 2008, 2009, 2010. Website also hosts a range of other ethical resources.
2	Canadian Psychiatric Association	Canadian Psychiatric Association (cpa-apc.org)	Has a code of ethics: The 1996 CMA Code of Ethics Annotated for Psychiatrists (first published 1980)
		Mexico, Central America and the Caribbean	
3	Barbados Association of Psychiatrists	No website found	Uses the code of a national general medical association. Reference Ghodse, H. (Ed.). (2011). International Perspectives on Mental Health. Cambridge: Royal College of Psychiatrists notes that it relies on the Barbados Medical Council Code of Conduct (2015)

4	Costa Rican Psychiatric Association	No website found	Has no website or any other online presence (although it is possible that ethical aspects of practice do exist)
5	Cuban Society of Psychiatry	Psiquiatría (sld.cu)	Has a website but does not refer to any ethical resources
6	Dominican Society of Psychiatry	No website found	Has no website or any other online presence (although it is possible that ethical aspects of practice do exist)
7	Salvadorean Association of Psychiatry	http://psiquiatraselsalvador.com/	Has a website but does not refer to any ethical resources
8	Guatemalan Psychiatric Association	http://asociacionpsiquiatricadeguatemala.org/	States that ethics is central to psychiatric practice but does not elaborate. States it has a code of ethics but no further information.
9	Jamaica Psychiatric Association	No website found. Facebook page.	Uses the code of a national general medical association. Reference Ghodse, H. (Ed.). (2011). International Perspectives on Mental Health. Cambridge: Royal College of Psychiatrists notes that it relies on the Jamaica Medical Council Code of Conduct (2015)
10	Honduran Society of Psychiatry	Asociación Hondureña de Psiquiatría (bvs.hn)	Has a website but does not refer to any ethical resources
11	Mexican Psychiatric Association	Asociación Psiquiátrica Mexicana A.C. (psiquiatrasapm.org.mx)	Has a website but does not refer to any ethical resources
12	Mexican Society of Neurology and Psychiatry	No website found. Facebook page	Has no website or any other online presence (although it is possible that ethical aspects of practice do exist)
13	Nicaraguan Psychiatric Association	No website found	Has no website or any other online presence (although it is possible that ethical aspects of practice do exist)
14	Panamanian Society of Psychiatry	Psipanama - Sociedad Panameña de Psiquiatría	Has a website but does not refer to any ethical resources

15	Puerto Rican Society of Psychiatry	https://nesile.tripod.com/prpsenglish.html	Uses the code of a universal or regional psychiatric organisation [Is a distinct branch of APA and therefore follows that code].
		Northern South America	
16	Colombian Association of Psychiatry	Asociación Colombiana de Psiquiatria – ACP	Has a code of ethics: 'Ethical principles and deontological code Colombian association of psychiatry' (2008). Available at: COMITÉ DE ÉTICA – Asociación Colombiana de Psiquiatria
17	Ecuadorian Association of Psychiatry	Asociación de psiquiatría núcleo del azuay – psiquiatría ecuador (psiquiatriaecuador.com)	Has a website but does not refer to any ethical resources
18	Peruvian Psychiatric Association	Asociacion Psiquiatrica Peruana (app.org.pe)	Has a code of ethics. Available at: https://www.app.org.pe/codigoetica.html
19	Venezuelan Society of Psychiatry	No website found. Facebook page and twitter	Has no website or any other online presence (although it is possible that ethical aspects of practice do exist)
		Southern South America	
20	Argentinean Association of Psychiatrists (AAP)	Asociación Argentina de Psiquiatras (aap.org.ar)	Has a code of ethics (no obvious date) available at: Asociación Argentina de Psiquiatras (aap.org.ar)
21	Association of Argentinean Psychiatrists (APSA)	Asociación de Psiquiatras Argentinos (APSA)	Has a website but does not refer to any ethical resources
22	Foundation for Interdisciplinary Investigation of Communication (FINTECO) (Argentina)	No website found	Has no website or any other online presence (although it is possible that ethical aspects of practice do exist)
23	Bolivian Society of Psychiatry	INICIO Bolivian Society of Psychiatry (weebly.com)	Has a website but does not refer to any ethical resources

24	Brazilian Association of Psychiatry	Associação Brasileira de Psiquiatria - ABP	Uses the code of a national general medical association. Website states: 'We inform that the Brazilian Association of Psychiatry (ABP) is a Specialty Society of the Brazilian Medical Association (AMB) and follows the Code of Ethics of the Federal Council of Medicine (CFM), which is the regulator of the professional conduct of the doctor.'
25	Psychiatric Association of Rio de Janeiro State (Brazil)	Aperj (aperjrio.org.br)	Has a website but does not refer to any ethical resources
26	Psychiatric Association of Rio Grande do Sul – APRS (Brazil)	No website found. Facebook page	Has no website or any other online presence (although it is possible that ethical aspects of practice do exist)
27	Society of Neurology, Psychiatry and Neurosurgery (Chile)	Sonepsyn	Has a website but does not refer to any ethical resources
28	Paraguayan Society of Psychiatry	Sociedad Paraguaya de Psiquiatría - Inicio (psiquiatriaparaguaya.org)	Has a website but does not refer to any ethical resources
29	Society of Psychiatry of Uruguay	Sociedad de Psiquiatría del Uruguay (spu.org.uy)	Has a website but does not refer to any ethical resources
		Europe	
		Western Europe	
30	Austrian Society for Psychiatry and Psychotherapy	ÖGPP — (oegpp.at)	States that ethics is central to psychiatric practice but does not elaborate (includes a history and ethics section but no further information).
31	Royal Society of Mental Medicine of Belgium	Société Royale de Médecine Mentale de Belgique	Has a website but does not refer to any ethical resources

32	Flemish Psychiatric Association (Belgium)	Vlaamse Vereniging voor Psychiatrie VVP Home (vvp-online.be)	Has an ethics (professional practice) committee but no code cited
33	French Association of Psychiatrists in Private Practice (AFPEP)	AFPEP-SNPP Association Française des Psychiatres d'Exercice Privé & Syndicat National des Psychiatres Privés (afpep-snpp.org)	Has a Charter of Psychiatry (1999), first adopted 1999. Available at: Charte de la psychiatrie
34	French Association of Psychiatry	Fédération française de psychiatrie (fedepsychiatrie.fr)	Has a website but does not refer to any ethical resources
35	French Psychiatric Information Society	Accueil - La Société de l'Information Psychiatrique (sphweb.fr)	Has a website but does not refer to any ethical resources
36	International Society of Psychopatholog y of Expression (France)	Société Française de Psychopathologie de l'Expression et d'Art-thérapie (sfpeat.com)	Has a website but does not refer to any ethical resources
37	Medical Psychologic Society (France)	No website found	Has no website or any other online presence (although it is possible that ethical aspects of practice do exist)
38	The Psychiatric Evolution (France)	L'Évolution psychiatrique – Cahiers de psychologie clinique et de psychopathologie (levolutionpsychiatrique.fr)	Has a website but does not refer to any ethical resources
39	German Association for Psychiatry, Psychotherapy and Psychosomatics	DGPPN Gesellschaft	States that ethics is central to psychiatric practice but does not elaborate. Ethics is listed as a common value that guides all work/guidelines.

40	College of Psychiatry of Ireland	The College of Psychiatrists of Ireland (irishpsychiatry.ie)	Has a code of ethics (2019) available here: Professional Ethics for Psychiatrists (irishpsychiatry.ie)
41	Luxembourguese Society of Psychiatry, Neurology and Psychotherapy	No website found	Has no website or any other online presence (although it is possible that ethical aspects of practice do exist)
42	Netherlands Psychiatric Association	Nederlandse Vereniging voor Psychiatrie (nvvp.net)	Has a code of ethics (2010) available here: Beroepscode (nvvp.net) First published 2003
43	Swiss Society of Psychiatry	Home – SGPP (psychiatrie.ch)	Has a website but does not refer to any ethical resources
44	The Royal College of Psychiatrists (UK)	Royal College of Psychiatrists (rcpsych.ac.uk)	Has a code of ethics (2014) available here: college-report-cr186.pdf (rcpsych.ac.uk)
		Northern Europe	
45	Danish Psychiatric Association	Dansk Psykiatrisk Selskab - Dansk Psykiatrisk Selskab (dpsnet.dk)	Has an ethics committee but no code cited. Role listed as tasked with assisting the Board in ethical matters by drafting written responses for approval by the DPS Board or by drafting ethical guidelines.
46	The Estonian Psychiatric Association	Eesti Psühhiaatrite Selts – Estonian Psychiatric Association	Has a code of ethics (no obvious date) available here: Eetikajuhised – Eesti Psühhiaatrite Selts (psy.ee)
47	Finnish Psychiatric Association	Etusivu - Suomen Psykiatriyhdistys	Uses the code of a universal or regional psychiatric organization. The 'values of the Finnish Psychiatric Association' section on ethics states: 'Ethical action is based on taking responsibility for what is right and appropriate in helping each patient. The psychiatrist should be aware of the principles of the Madrid Declaration of the World Psychiatric Association (WPA).' It also has an ethics committee listed.
48	Icelandic Psychiatric Association	Geðhjálp (gedhjalp.is)	Uses guidelines or resources on specific ethical issues in psychiatry but has no code. Has a human rights portal to learn about patient's human rights and ethics: Forsíða » Réttindagátt.is (rettindagatt.is)

49	Latvian Psychiatric Association	Sākums (psihiatruasociacija.lv)	Has a website but does not refer to any ethical resources
50	Lithuanian Psychiatric Association	Naujienos | Lietuvos Psichiatrų Asociacija (psichiatrija.lt)	Uses the code of a universal or regional psychiatric organisation. Dedicated page on website for 'professional ethics' including national codes on medicines marketing, and forensic services. This page also links to WPA codes and APA code. Profesinė etika | Lietuvos Psichiatrų Asociacija (psichiatrija.lt)
51	Norwegian Psychiatric Association	Norsk psykiatrisk forening (legeforeningen.no)	Uses the code of a universal or regional psychiatric organisation. Notes it is a specialist medical unit of the Norwegian Medical Association, which has a code of ethics for doctors (2015)
52	Swedish Psychiatric Association	Svenska Psykiatriska Föreningen – (svenskpsykiatri.se)	Has a website but does not refer to any ethical resources. Notes the association is both a specialist association and the section of the Swedish Society of Medicine, which does not appear to have a code.
Southern Europe			
53	Albanian Psychiatric Association	Lidhja e Psikiatrisë Shqiptare (lpsh-al.org)	Uses guidelines or resources on specific ethical issues in psychiatry but has no code (e.g. congress and educational events organized on ethics in psychiatry).
54	Cyprus Psychiatric Association	No website	Has no website or any other online presence (although it is possible that ethical aspects of practice do exist)
55	Hellenic Psychiatric Association	Ελληνική Ψυχιατρική Εταιρεία (psych.gr)	Has a website but does not refer to any ethical resources.
56	Hellenic Society of Neurology and Psychiatry	Η εταιρεια - ελληνικη νευρολογικη εταιρεια (enee.gr)	Has a website but does not refer to any ethical resources.
57	Israeli Psychiatric Association	No website	Uses the code of a national general medical association. Relies on Israel Medical Association Ethics Board Rules and Position Papers (which incorporates the Code of Ethics, 2018) EthicalCode2018.pdf (ima.org.il)
58	Italian Association for Research in Schizophrenia	No website	Has no website or any other online presence (although it is possible that ethical aspects of practice do exist)

59	Italian Psychiatric Association	Società Italiana di Psichiatria	Has a website but does not refer to any ethical resources. Does state it is affiliated to WPA.
60	Portuguese Association of Psychiatry	SPPSM | Sociedade Portuguesa de Psiquiatria e Saúde Mental	Has a website but does not refer to any ethical resources.
61	Portuguese Society of Psychiatry and Mental Health	Apperas to be part of the Portuguese Association of Psychiatry (see line 60 above)	Has a website but does not refer to any ethical resources.
62	Spanish Association of Neuropsychiatry	AEN – Profesionales de Salud Mental	Has a website but does not refer to any ethical resources.
63	Spanish Society of Psychiatry	Sociedad Española de Psiquiatría (sepsiq.org)	Uses guidelines or resources on specific ethical issues in psychiatry but has no code. It has a Code of Good Practice, which is is focused on the society as an entity, not individual's practice and ethics. http://www.sepsiq.org/informacion/buenasPracticas
64	Psychiatric Association of Turkey	Türkiye Psikiyatri Derneği (TPD)	Has an ethics committee but no code cited. Ethics Committee tasked with reviewing ethical issues noting all decisions are recorded and kept.
65	Turkish Neuropsychiatric Society	Türk Nöro-Psikiyatri Derneği (turknoropsikiyatri.org)	Has a website but does not refer to any ethical resources.
Central Europe			
66	Psychiatric Association of Bosnia-Herzegovina	Udruženje / Udruga psihijatara u Bosni i Hercegovini – (upubih.com)	Has a website but does not refer to any ethical resources.
67	Bulgarian Psychiatric Association	Начало | Българска Психиатрична Асоциация - БПА (bpabg.com)	Has an ethics committee but no code cited.
68	Croatian Psychiatric Association	Hrvatsko psihijatrijsko društvo (psihijatrija.hr)	Has a website but does not refer to any ethical resources, only clinical guidelines.

69	Czech Psychiatric Association	Psychiatrická společnost ČLS JEP a Česká psychiatrická společnost z.s. (psychiatrie.cz)	Has a website but does not refer to any ethical resources
70	Hungarian Psychiatric Association	Magyar Pszichiátriai Társaság On-line (mptpszichiatria.hu)	Has a code of ethics (2010 listed under company documents (accessible to members only).
71	Macedonian Psychiatric Association (Republic of North Macedonia)	No website	Has no website or any other online presence (although it is possible that ethical aspects of practice do exist)
72	Montenegrin Psychiatric Association	No website	Has no website or any other online presence (although it is possible that ethical aspects of practice do exist)
73	Polish Psychiatric Association	Polskie Towarzystwo Psychiatryczne. (psychiatria.org.pl)	Has a website but does not refer to any ethical resources
74	Association of Free Psychiatrists of Romania	No website	Has no website or any other online presence (although it is possible that ethical aspects of practice do exist)
75	Romanian Association of Psychiatry and Psychotherapy	e-Psihiatrie.ro – Asociatia Romana de Psihiatrie si Psihoterapie (e-psihiatrie.ro)	Uses guidelines or resources on specific ethical issues in psychiatry but has no code. States that it is a member of the European Psychiatric Association (EPA) and follows that code. Further available are a range of legislative articles related confidentiality and patient rights.
76	Serbian Psychiatric Association	http://en.ups-spa.org	Has a website but does not refer to any ethical resources
77	Association of Psychiatric Institutions of Serbia - UPUS	No website	Has no website or any other online presence (although it is possible that ethical aspects of practice do exist)

78	Slovak Psychiatric Association	Slovenská psychiatrická spoločnost SLS (psychiatry.sk)	Has an ethics committee but no code cited: Ethics Committee. Includes links to 2015 'ethics opinions' that are only available Slovakian.
79	Psychiatric Association of Slovenia	Združenje psihiatrov Slovenije (zpsih.si)	Has a website but does not refer to any ethical resources. Has a range of professional guidelines for psychiatrists but none covering purely ethics. Is part of the Slovenian Medical Association.
	Eastern Europe		
80	Armenian Psychiatric Association	ՀԱՅԿԱԿԱՆ ՀՈԳԵԲՈՒԺԱԿԱՆ ԱՍՈՑԻԱՑԻԱ - Գլխավոր (apnet.am)	Has a code of professional ethics for psychiatrists (no obvious date)
81	Azerbaijan Psychiatric Association	No website	Has no website or any other online presence (although it is possible that ethical aspects of practice do exist). Eg. In Ghodse, H. (Ed.). (2011). International Perspectives on Mental Health. Cambridge: Royal College of Psychiatrists it states that Azerbaijan Psychiatric Association has worked on project with WPA and others to translate ICD10, ethical guidelines for psychiatrists and Madrid Declaration into the Azerbaijan language and distribute these.
82	Belarusian Psychiatric Association	No website	Has no website or any other online presence (although it is possible that ethical aspects of practice do exist).
83	Society of Georgian Psychiatrists	No website	Has no website or any other online presence (although it is possible that ethical aspects of practice do exist).
84	Kazakh Association of Psychiatrists & Narcologists	No website found	Has no website or any other online presence (although it is possible that ethical aspects of practice do exist).
85	The Association of specialists working in the field of mental health (Kazakhstan) (Ad-Hoc)	No website found	Has no website or any other online presence (although it is possible that ethical aspects of practice do exist).

86	Kyrgyz Psychiatric Association	No website found	Has no website or any other online presence (although it is possible that ethical aspects of practice do exist).
87	League for Mental Health from Republic of Moldova	No website found	Has no website or any other online presence (although it is possible that ethical aspects of practice do exist).
88	Independent Psychiatric Association of Russia	Об ассоциации - npar.ru	Has a website but does not refer to any ethical resources
89	Russian Society of Psychiatrists	Российское Общество Психиатров (РОП) (psychiatr.ru)	Has a code of ethics (1994) available here: Кодекс профессиональной этики психиатра - РОП (psychiatr.ru) Also has a code of ethics for forensic psychiatric examination.
90	Association of Neurologists, Psychiatrists and Narcologists of Ukraine	Асоціація неврологів, психіатрів та наркологів України (inpn.org.ua)	Has an ethics committee but no code cited (ethics and deontology commission)
91	Ukrainian Psychiatric Association	Асоціація психіатрів України — Асоціація Психіатрів України (ukrpsychiatry.org)	Has a website but does not refer to any ethical resources
92	Association of Psychiatrists of Uzbekistan	Uzbekistan Psychiatric Association (psychiatry.uz)	Has a website but does not refer to any ethical resources
		Africa and the Middle East	
		Northern Africa	
93	Algerian Psychiatric Association	Société Algérienne de Psychiatrie (S.A.P) (sapsy-dz.com)	Has a website but does not refer to any ethical resources
94	Egyptian Psychiatric Association	النفسي للطب المصرية الجمعية - الرئيسية الصفحة (epassociation.net)	Has a website but does not refer to any ethical resources

95	Libyan Association of Psychiatry, Neurology and Neurosurgery	No website	Has no website or any other online presence (although it is possible that ethical aspects of practice do exist).
96	Moroccan Society of Psychiatry	No website	Has no website or any other online presence (although it is possible that ethical aspects of practice do exist).
97	Sudanese Association of Psychiatrists	No website	Has no website or any other online presence (although it is possible that ethical aspects of practice do exist).
98	Tunisian Society of Psychiatry	No website	Has no website or any other online presence (although it is possible that ethical aspects of practice do exist).
Middle East			
99	Arab Gulf Psychiatric Association	No website	Has no website or any other online presence (although it is possible that ethical aspects of practice do exist).
100	Emirates Society of Mental Health	No website	Has a website but does not refer to any ethical resources. Under umbrella of Emirates Medical Association (EMA). EMA lists ethics as core value but has no code
101	Iraqi Society of Psychiatrists	No website	Has no website or any other online presence (although it is possible that ethical aspects of practice do exist).
102	Jordan Association of Psychiatrists	No website. Facebook group	Has no website or any other online presence (although it is possible that ethical aspects of practice do exist). States it is a division of the Jordan Medical Syndicate.
103	Kuwait Psychiatric Association	KMA (Kuwait Medical Association)	Has a website but does not refer to any ethical resources. Uses the charter of a national general medical association. It is part of the Kuwait Medical Association, which also has a Professional Ethics Association which states it isusing the International Islamic Medical Charter as a model as a charter for Kuwait.
104	Lebanese Psychiatric Society	Lebanese Psychiatric Society (lpsonline.org)	Uses guidelines or resources on specific ethical issues in psychiatry but has no code. Ethics included as part of mission statement. Has a section of ethics in psychiatry guidelines on website covering psychotherapy, substance use disorder, child and adolescent, and challenges in the internet era.

105	Palestinian Psychiatric Association	No website	Has no website or any other online presence (although it is possible that ethical aspects of practice do exist).
106	Saudi Psychiatric Association	Saudi Psychiatric Association	States that ethics is central to psychiatric practice but does not elaborate.The importance of ethics included as part of a statement on practice and standards.
107	Syrian Arab Association of Psychiatrists	http://www.psysyr.org/	Has a website but does not refer to any ethical resources. Website not current. Syrian Association for Mental Health (syriasamh.com) (including psychiatrists, psychologists and social workers) has been established but has no code of ethics.
108	Yemen Psychiatrists & Neurologists Association	No website.	Has no website or any other online presence (although it is possible that ethical aspects of practice do exist). E.g in Ghodse, H. (Ed.). (2011). International Perspectives on Mental Health. Cambridge: Royal College of Psychiatrists it suggests the organization advocates for establishment of formal code of ethics and mental health act at great personal and emotional expense, but no further information about this was found.
Central and Western Africa			
109	Ghana Psychiatric Association	Facebook page. Website under construction	Has no website or any other online presence (although it is possible that ethical aspects of practice do exist).
110	Association of Psychiatrists in Nigeria	Association of Psychiatrists of Nigeria (apn.org.ng)	States that ethics is central to psychiatric practice but does not elaborate. Mission includess to maintain a high standard of professional ethics, conduct and practice.
111	Society of Psychopathology and Mental Hygiene of Dakar (Senegal)	No website	Has no website or any other online presence (although it is possible that ethical aspects of practice do exist).
Eastern and Southern Africa			

112	Botswana Psychiatric Association	No website. Facebook page.	Has no website or any other online presence (although it is possible that ethical aspects of practice do exist).
113	Ethiopian Psychiatric Association (Zone 11 to 14 – GA Berlin 2017)	EPA – Ethiopian Psychiatric Association (epanets.org)	Uses the code of a universal or regional psychiatric organization. Mission includes ethics, and states it has developed ethical guidelines for psychiatric practice, including the Madrid Declaration (1996) and relies on UN conventions etc.
114	Kenya Psychiatric Association	Kenya Psychiatric Association (kenyapsychiatrist.org)	Uses the code of a universal or regional psychiatric organization. Mission and core values include ethics but no code. Notes it is also a partner of American Psychiatric Association, and refers to code.
115	Mauritius Psychiatric Association	No website	Has no website or any other online presence (although it is possible that ethical aspects of practice do exist).
116	Mozambican Association of Psychiatry and Mental Health	No website	Has no website or any other online presence (although it is possible that ethical aspects of practice do exist).
117	South African Society of Psychiatrists	South African Society of Psychiarists | Sasop	Uses guidelines or resources on specific ethical issues in psychiatry but has no code Has a range of position statements, some with ethical relevance. Also states that ethics is central to psychiatric practice but does not elaborate.
118	Uganda Psychiatric Association	www.ugandapsychiatricassociation.com	States that ethics is central to psychiatric practice but does not elaborate. Ethics is part of mission statement: To promote the image of psychiatry in Uganda guided by high ethical practices.
119	Zimbabwe College of Psychiatrists (Ad-Hoc)	No website	Has no website or any other online presence (although it is possible that ethical aspects of practice do exist).
120	Zambia Psychiatric Association (Ad-Hoc)	No website. Facebook page	Has no website or any other online presence (although it is possible that ethical aspects of practice do exist).
	Asia and Australasia		

Central and Western Asia			
121	Afghanistan National Psychiatrist Association (ANPA)	No website. Facebook Page	Has no website or any other online presence (although it is possible that ethical aspects of practice do exist).
122	Iranian Psychiatric Association	Iranian Psychiatric Association (psychiatrist.ir)	Has a website but does not refer to any ethical resources.
123	Pakistan Psychiatric Society	Pakistan Psychiatric Society (ppspk.com)	States that ethics is central to psychiatric practice but does not elaborate. Teaching of ethics is mentioned under educational mission.
Southern Asia			
124	Bangladesh Association of Psychiatrists	Bangladesh Association of Psychiatrists - (bapbd.org)	States that ethics is central to psychiatric practice but does not elaborate. Ethics is is mentioned under mission and values To promote ethical standards in the practice of psychiatry in Bangladesh.
125	Indian Association for Social Psychiatry	Indian Association for Social Psychiatry (iasp.org.in)	Has a website but does not refer to any ethical resources
126	Indian Psychiatric Society	Indian Psychiatric Society	Has a website but does not refer to any ethical resources. Literature [*Nallur DG. The need for "code of practice" as a supplement to Mental Healthcare Act 2017. Indian journal of psychiatry. 2019 Apr;61(Suppl 4):S798*] confirms that the The Indian Psychiatric Society (IPS), in its 1989 annual conference, formed a committee to recommend a code of ethics, particularly for psychiatrists. And that, more recently, in December 2018, the Ethics Subcommittee of the IPS published version 1.3 of the code of ethics for psychiatrists in India. This does not appear on the website and attempts to contact the IPS to seek further information were not successful.
127	Indonesian Psychiatric Association	Indonesian Psychiatric Association | PDSKJI.org	Has a website but does not refer to any ethical resources

128	Malaysian Psychiatric Association	Malaysian Psychiatric Association - MPA (psychiatry-malaysia.org)	States that ethics is central to psychiatric practice but does not elaborate. States that the MPA is concerned with several issues including an Ethical Code of members but there is no further information. Further states it is a member of the Asia Federation of Psychiatric Associations (AFPA), which also has no available code.
129	Myanmar Medico-Psychological Society	Mental Health Society | Myanmar Medical Association (MMA) (mmacentral.org)	Has a website but does not refer to any ethical resources. Appears to be part of the Myanmar Medical Association, but neither has a code of ethics.
130	Psychiatrists' Association of Nepal	No website. Facebook group	Uses the code of a universal or regional psychiatric organization. Confirmed via literature [Upadhyaya, K., & Joshi, D. (2014). Ethics in mental Health in resource poor setting: experiences from Nepal. Journal of Psychiatrists' Association of Nepal, 1(1), 5-10.]
131	Philippine Psychiatric Association	Philippine Psychiatric Association	Has an ethics committee but no code cited**.**
132	Singapore Psychiatric Association	Singapore Psychiatric Association (singaporepsychiatry.org.sg)	Uses guidelines or resources on specific ethical issues in psychiatry but has no code The Association training guide (*Mastering Psychiatry, core textbook for undergraduates, 2015*) provides an overview ethical obligations.
133	Sri Lanka College of Psychiatrists	SL College of Psychiatrists (slcpsych.lk)	Has a website but does not refer to any ethical resources.
133	The Psychiatric Association of Thailand	วารสาร สมาคมจิตแพทย์แห่งประเทศไทย The Psychiatric Association of Thailand - (psychiatry.or.th)	Has a website but does not refer to any ethical resources.
		Eastern Asia	
134	Mental Health Association of Cambodia (MHAC)	No website	Has no website or any other online presence (although it is possible that ethical aspects of practice do exist).
135	Chinese Society of Psychiatry	csp.cma.org.cn	Has a website but does not refer to any ethical resources.

136	The Hong-Kong College of Psychiatrists (China)	The Hong Kong College of Psychiatrists - Home (hkcpsych.org.hk)	Has an ethics committee but no code cited**.**
137	The Japanese Society of Psychiatry and Neurology	The Japanese Society of Psychiatry and Neurology (jspn.or.jp)	Has a code of ethics (2014) available here: Code of Ethics for Psychiatrists_2015 (jspn.or.jp).<br><br>Note that the International Mental Health Professionals Japan also has a code of ethics: Code of Ethics - International Mental Health Professionals Japan (imhpj.org)
138	Korean Neuropsychiatric Association	대한신경정신의학회 (knpa.or.kr)	States that ethics is central to psychiatric practice but does not elaborate.
139	Mongolian Mental Health Association	No website	Has no website or any other online presence (although it is possible that ethical aspects of practice do exist).
140	Taiwanese Society of Psychiatry	www.sop.org.tw	States that ethics is central to psychiatric practice but does not elaborate. Hosts guidelines on stigma reduction.
		Australasia and the South Pacific	
141	The Royal Australian and New Zealand College of Psychiatrists	Royal Australian and New Zealand College of Psychiatrists | RANZCP	Has a code of ethics (2018). Available at: RANZCP Code of Ethics.<br>Previous editions 1992, 1998, 2004, 2010.
142	Papua New Guinea Psychiatric Association	No website	Has no website or any other online presence (although it is possible that ethical aspects of practice do exist).
